# Mycobacterium tuberculosis Rv0790c inhibits the cellular autophagy at its early stage and facilitates mycobacterial survival

**DOI:** 10.3389/fcimb.2022.1014897

**Published:** 2022-11-09

**Authors:** Jun Fang, Chunsheng Dong, Sidong Xiong

**Affiliations:** Jiangsu Key Laboratory of Infection and Immunity, Institutes of Biology and Medical Sciences, Soochow University, Suzhou, China

**Keywords:** *Mycobacterium tubercul*osis, Rv0790c, macrophage, autophagy, mTOR

## Abstract

Rv0790c is predicted to be a conserved hypothetical protein encoded by *Mycobacterium tubercul*osis (*Mtb*). However, its function in *Mtb* infection remains largely unknown. In this study, we found that Rv0790c promoted bacillary survival of *M. smegmatis* (*Ms*), both *in vitro* and *in vivo*. The bacillary burden of *Ms* exogenously expressing Rv0790c increased, whereas in Rv0790c-knockouts the bacillary burden decreased in infected macrophages. Multiple cellular processes were analyzed to explore the underlying mechanisms. We found that neither inflammatory regulation nor apoptotic induction were responsible for the promotion of bacillary survival mediated by Rv0790c. Interestingly, we found that Rv0790c facilitates mycobacterial survival through cellular autophagy at its early stage. Immunoprecipitation assay of autophagy initiation-related proteins indicated that Rv0790c interacted with mTOR and enhanced its activity, as evidenced by the increased phosphorylation level of mTOR downstream substrates, ULK-1, at Ser^757^ and P70S6K, at Thr^389^. Our study uncovers a novel autophagy suppressor encoded by mycobacterial Rv0790c, which inhibits the early stage of cellular autophagy induction upon *Mtb* infection and takes an important role in maintaining intracellular mycobacterial survival. It may aid in understanding the mechanism of *Mtb* evasion of host cellular degradation, as well as hold the potential to develop new targets for the prevention and treatment of tuberculosis.

## Introduction

Tuberculosis (TB) is a chronic disease caused by *Mycobacterium tuberculosis* (*Mtb*), affecting 2 billion people, which results in 1.5 million deaths every year (WHO, 2021). Compared with extracellular bacteria, *Mtb* can escape host surveillance and successfully replicate in immune cells. The emergence of multidrug-resistant strains makes TB treatment even more difficult (Wu et al., 2020). Therefore, novel strategies for the prevention and treatment of TB are urgently needed.

Macrophages, especially alveolar macrophages, are the first line of defense against *Mtb* infections (Cohen et al., 2018). During an *Mtb* infection, macrophages eliminate invading pathogens through multiple cellular processes, including autophagy, an intracellular homeostatic process that degrades damaged cell components and organelles *via* lysosomal fusion through a double-membrane vesicle (Bell et al., 2021). Usually, this process is initiated by the inhibition of mTOR complexes, followed by activation of the ULK complex and downstream PI3K-Beclin complex. Many autophagy-related proteins (ATGs) participate in autophagosome formation, including the ATG5/ATG12/ATG16 complex and LC3-II. Finally, the substrate-containing autophagosomes fuse with lysosomes to degrade the cargo (Odle et al., 2020). Several proteins, such as that of the Rab7 and Atg8 family (Lorincz and Juhasz, 2020), are required at the fusion stage. In addition to maintaining cell homeostasis, autophagy is also involved in the elimination of invading viruses and bacterial pathogens like *Mtb*. Emerging evidence suggests that targeting macrophage autophagy can be employed to develop anti-TB drugs. For example, induction of macrophage autophagy by CLEC4E, in combination with TLR4 agonists, plays a vital role in inhibiting *Mtb* survival (Pahari et al., 2020). Stimulation of TLR7 with imiquimod induces autophagy and controls *Mtb* growth in mouse macrophages (Lee et al., 2020). Ornithine, a urea cycle metabolite, suppresses *Mtb* growth *via* the enhancement of AMPK-mediated autophagy in macrophages (Sivangala Thandi et al., 2020). Meanwhile, *Mtb* has evolved a diverse range of mechanisms to evade host cell elimination. It has been reported that several *Mtb* virulence factors are associated with autophagy suppression. For instance, *Mtb* PknG inhibits autophagy by interacting with Rab14 to block Rab14-GTP hydrolysis (Ge et al., 2021), HBHA decreases autophagy to promote its survival and replication (Zheng et al., 2017), while RipA suppresses autophagy initiation by activating the PI3K-AKT-mTORC1 signaling cascade by interacting with TLR4 (Shariq et al., 2021). Hence, identifying new *Mtb* proteins that trigger macrophage autophagy will help us understand and develop more effective anti-TB strategies by modulating autophagy.

Rv0790c is a conserved hypothetical protein encoded by *Mtb.* The exact function of Rv0790c in *Mtb* infection is poorly understood, however, some studies indicated that the Rv0790c level was upregulated after antibiotic treatment (Denkin et al., 2005; Mehra and Kaushal, 2009) and a genome-wide *Mtb* transposon mutant library implied that Rv0790c might affect bacillary growth (Rengarajan et al., 2005). In this study, we found that Rv0790c inhibited autophagy, but not inflammation and apoptosis, in *Mtb* infected macrophages and facilitated bacterial survival. This inhibitory effect relies on its interaction with mTOR and its subsequent augmentation; our study provides a novel therapeutic target for anti-TB drug development.

## Materials and methods

### Mice, cells and bacteria

Female C57BL/6 mice, aged 6–8 weeks, were purchased from the Experimental Animal Center of the Chinese Academy of Sciences (Shanghai, China) and maintained under specific pathogen-free conditions at Soochow University. All experimental animal procedures were performed in accordance with the guidelines for the Care and Use of Laboratory Animals (Ministry of Health, China, 1998). The guidelines were approved by the ethics committee of Soochow University. HEK293T cells and the murine macrophage cell line, RAW264.7, were cultured in Dulbecco’s modified Eagle’s medium (DMEM, Hyclone), supplemented with 1% penicillin-streptomycin (Invitrogen) and 10% fetal bovine serum (FBS, Hyclone). Human monocytic THP-1 cells were cultured in Roswell Park Memorial Institute medium (RPMI, Hyclone) supplemented with 1% penicillin-streptomycin and 10% FBS. For THP-1-derived macrophages, 40 ng/mL phorbol 12-myristate 13-acetate (PMA, Sigma-Aldrich) was used to stimulate THP-1 cells for three days. Cells were cultured in a humidified incubator at 37°C and 5% CO_2_. The *M. smegmatis* (*MS*) strain mc^2^155 was grown in Luria-Bertani medium supplemented with 0.05% Tween-80 (Sigma). *H37Rv* and *H37RvΔRv0790c* were purchased from Gene Optimal Inc., Shanghai, and grown in Middlebrook 7H9 broth medium (Becton Dickinson) supplemented with 10% ADC (Becton Dickinson), 0.5% glycerol (Sigma), and 0.05% Tween-80 at 37°C.

### Plasmid construction

The Rv0790c gene was amplified from *H37Rv* genomic DNA using specific primers (sense- 5′-CCCAAGCTTATGACGCTAGCCAACAATGGAAC-3′; antisense- 5′CGC GGATCCTCACAGTCGGTGGGTTGCG-3′) and cloned into pFlag-CMV2, using HindIII and BamHI sites, and named pFlag-CMV2-Rv0790c. The Rv0790c gene was amplified using specific primers (sense- 5′-CGCGGATCCATGACGCTAGCCAA CAATGGAAC-3′; antisense-5′-CAGAAGCTTCTACTTGTCGTCATCGTCTTTGT A GTCCAGTCGGTGGGTTGCGTCG-3′) and cloned into the pMV261 vector, using BamHI and HindIII sites with a C-terminal FLAG-tag, generating pMV261-Rv0790c. The Rv0790c gene was amplified using specific primers (sense- 5′-CTCGAGATG ACGCTAGCCAACAATGGAAC-3′; antisense- 5′-GTTAACCTACTTGTCGTCATC GTCTTTGTAGTCCAGTCGGTGGGTTGCGTCG-3′) and cloned into the pMSCV-eGFP retroviral vector using the XhoI and HpaI sites to generate pMSCV-eGFP-Rv0790c.

### Generation of recombinant *MS_Rv0790c* and the stable cell line, RAW-Rv0790c

Plasmid pMV261-Rv0790c and empty vector pMV261 were electroporated into *MS* strain mc^2^155, generating *MS_Rv0790c* and *MS_WT* after kanamycin (50 µg/mL) selection, respectively. The expression of Rv0790c in single colonies was confirmed by western blotting with anti-Flag antibody in harvested *MS_Rv0790c.* HEK293T cells were co-transfected with pMSCV-eGFP-Rv0790c and the packaging vector, pCL-Ampho, using Lipofectamine3000 (Invitrogen). The cell culture supernatant was harvested 48 h and 72 h post-transfection, and filtered through a 0.45-μm pore size filter. RAW264.7 cells were infected with viral supernatants containing 10 μg/mL polybrene (Sigma) and then centrifuged at 1500 × *g*, for 90 min, at 30°C, to improve infection efficiency. Cells were then cultured in fresh medium for 72 h, and GFP-positive cells were sorted by BD FACS Aria II flow cytometry (Becton Dickinson) and named RAW-Rv0790c. RAW-Vector cells were generated by infection with pMSCV-eGFP-packaged retrovirus as a control. The expression of Rv0790c in RAW-Rv0790c cells was verified using western blotting.

### XTT bacterial proliferation assays

In a 96-well plate 1×10^4^ of *MS_WT* and *MS_Rv0790c* were seeded, and the bacteria were incubated at 37°C for different time periods (0 h/12 h/24 h/48 h). Then, 20 μL of XTT working solution (Biolab, Beijing, KFS318) was added to each well and incubated at 37°C for 2 h. Bacterial proliferation was then evaluated by measuring the optical density at 450 nm.

### *MS* and *H37Rv* colony-forming unit (CFU) assay and mycobacterial survival detection

RAW264.7 or THP-1 cells were infected with *MS_WT* and *MS_Rv0790c* at a multiplicity of infection (MOI) = 20, or infected with *H37Rv* and *H37RvΔRv0790c* at MOI = 10. After 4 h of incubation, the cells were washed thrice with 1x PBS to remove extracellular bacteria and incubated with DMEM. The infected cells were washed thrice with 1x PBS at different time points and lysed with 1mL of sterile water, containing 0.05% Triton X-100. Lysates (50 µL) were plated on Middlebrook 7H10 agar. The *MS* CFU were counted 3 days post-plating, and the *Mtb* CFU were counted 21 days post plating. *In vivo*, mice were intranasally infected or intraperitoneally injected with 2×10^7^ *MS_WT* or *MS_Rv0790c* suspended in 30 µl PBS. Mice were sacrificed six days post-infection. The lung tissues were homogenized in 1mL PBS and 50 µL of the lysates were plated on 7H10 agar plates, and the bacterial CFU was counted 3–4 days post plating.

### Proteinase K digestion and bacterial plasmolysis assay

For the proteinase K digestion assay, equal amounts of *MS_WT* and *MS_Rv0790c* were collected and incubated with 100 μg/mL proteinase K at 37°C. PMSF (100 nM) was added at different time points to stop the reaction. Equal volumes of these bacteria were harvested and analyzed by western blotting, using an anti-Flag antibody for Rv0790c. For the bacterial plasmolysis assay, *MS_WT* and *MS_Rv0790c* bacterial cultures were ultrasonicated, and the cell lysates were centrifuged at 3,000 × *g* for 5 min. The supernatant was ultra-centrifuged at 27,000 × *g* for 40 min, after which they were identified as membrane-cytosol fractions and the pellets were identified as cell wall fractions. The expression of Rv0790c protein in each fraction was detected by western blotting using an anti-Flag antibody. GroEL, a mycobacterial cytosolic marker, was detected using an anti-GroEL antibody (Abcam, ab82592).

### Flow cytometry analysis

RAW264.7 cells were infected with *MS_WT* or *MS_Rv0790c* at a MOI of 10. The cells were then treated with trypsin 4 h post-infection, rinsed with 1x PBS containing 0.05% saponin, and incubated with an anti-LC3 antibody (CST, 3868) for 30 min. Finally, cells were incubated with anti-rabbit IgG488 (CST, 4412) for 30 min. The LC3 positive cells were acquired using a FACS Canto II flow cytometer (Becton Dickinson) and analyzed using FlowJo 7.6.1. For cellular apoptosis detection, RAW264.7 cells were seeded on a 24-well plate and infected with *MS_WT* or *MS_Rv0790c* at MOI = 10 for 48 h. Apoptotic cells were measured by a FACS Canto II flow cytometer using an Annexin V/PI kit (eBioscience) and analyzed using FlowJo 7.6.1.

### Western blotting

To detect the expression of the key proteins of autophagy, treated RAW264.7 cells were harvested and lysed in lysis buffer containing phosphatase and protease inhibitors for 30 min on ice. The protein concentration in the lysate was quantified using the BCA Protein Assay Kit (Beyotime Biotechnology). Equal amounts of protein from each sample were separated using SDS-PAGE and transferred to a PVDF membrane (Millipore). Subsequently, the membrane was blocked with 5% fat-free milk for 2 h at room temperature and incubated with primary antibodies overnight at 4°C. The next day, the membrane was incubated with horseradish peroxidase-conjugated goat anti-rabbit or anti-mouse IgG secondary antibody at room temperature for 1 h and detected using ECL (Thermo Pierce, Rockford, Illinois, USA). Chemiluminescence was visualized using Amersham Imager 600 (AI600; GE Healthcare). The primary antibodies used in this study were anti-LC3 (Sigma, AL7543), anti-mTOR (CST, 2983), anti-Phospho-P70S6K (Thr389, CST, 9234), anti-p70S6K (CST, 2708), anti-ULK (CST, 6439), anti-phospho-ULK (Ser757, CST, 14202), anti-GAPDH (ABclonal, A19056), anti-GroEL (Abcam, ab82592), anti-Flag (ABclonal, AE005), and anti-β-actin (ABclonal, AC026). The secondary antibodies used were HRP-conjugated anti-mouse or anti-rabbit IgG (Southern-Biotech, Beijing, China). For quantification, densitometry was performed using ImageJ software (version 1.6.0_20) to evaluate the intensity of the western blot band signals.

### Immunofluorescence and transmission electron microscopy

For IF, HEK293T cells were co-transfected with mCherry-GFP-LC3 and pFlag-CMV2-Rv0790c for 36 h. The cells were then treated with rapamycin for 4 h and the autophagosomes in the transfected cells were detected by confocal laser scanning microscopy (Nikon A1). HEK293T cells were co-transfected with GFP-LC3 and pFlag-CMV2-Rv0790c for 36 h. The cells were treated with rapamycin or together with chloroquine (CQ) for 4 h. Autophagosomes in the transfected cells were detected by confocal laser scanning microscopy. To examine Rv0790c and mTOR colocalization, HEK293T cells were transfected with pFlag-CMV2-Rv0790c for 36 h. The cells were then washed thrice with 1x PBS, fixed with 4% paraformaldehyde, and permeabilized with 0.05% Triton X-100. The cells were then blocked with 3% bovine serum albumin (BSA, Sigma) and incubated with anti-FLAG mouse antibody and anti-mTOR rabbit antibody at 4°C overnight. The next day, the cells were washed with 1× PBS thrice and incubated with DyLight 488-goat anti-mouse IgG (Jackson, 111-545-003) and DyLight 633-goat anti-rabbit IgG (Jackson, 111-605-144) for 30 min at room temperature, and the nuclei were stained with DAPI (Beyotime) for 15 min at room temperature. To determine the co-localization of mTOR and Rv0790c in the context of mycobacterial infection, RAW264.7 cells were infected with *MS_Rv0790c* (MOI = 10) for 4 h. Cell staining was performed as described above for confocal microscopy. For TEM, RAW264.7 cells were infected with *MS_WT* or *MS_Rv0790c* (MOI = 10) for 4 h. Each sample was dehydrated with increasing concentrations of ethanol, gradually adsorbed with the Epon-Araldite resin, and inserted into a straight resin. The samples were then heated to 80°C for 24 h. Ultrathin sections (70–80 nm) were stained with uranyl acetate and lead citrate, and then examined using a Hitachi 7650 TEM.

### TNF-α and IL-6 level detection

RAW264.7 cells were infected with *MS_WT* or *MS_Rv0790c* at an MOI of 10 for 24 h. The cells were harvested for RT-PCR and the culture supernatants were harvested for ELISA. Total cellular RNA was extracted using TRIzol reagent (Invitrogen), according to the manufacturer’s protocol. cDNA was synthesized using a reverse transcription kit (Takara). The relative mRNA levels of cytokines were detected by RT-PCR amplification using an ABI Q6 instrument (Applied Biosystems), and the following specific primer pairs were used: GAPDH (sense- 5′-GAGCCAAACGGGTCATCA TCT-3′; antisense- 5′-GAGGGGCCATCCACAGTCTT-3′, TNF-α (sense- 5′-TCTTCT CGAACCCCGAGTGA-3′; antisense- 5′-CCTCTGATGGCACCACCA-3′), IL-6 (sense-5′-AGGAGACTTGCCTGGTGAAA-3′; antisense-5′-CAGGGGTGGTTATT G CATCT-3′). Gene expression was analyzed using the 2^−△△CT^ method (Chiang et al., 2012; Salvador-Martin et al., 2020). TNF-α and IL-6 protein levels were determined using ELISA kits (Invitrogen), following the manufacturer’s protocols.

### Immunoprecipitation assay

HEK293T cells were transfected with plasmids containing the pFlag-CMV2 vector or pFlag-CMV2-Rv0790c for 36 h. The cells were then washed with pre-cooled 1x PBS and lysed in western and IP lysis buffer (Beyotime) containing 100 μM PMSF (Beyotime). Cell lysates were collected and incubated on a shaker with 25 μL EZviewTM Red Anti-Flag Affinity Gel (Sigma) at 4°C overnight. The beads were eluted with 1x loading buffer after washing five times with lysis buffer, and boiled for 15 min before western blotting analysis. Immunoprecipitation assay in RAW-Vector and RAW-Rv0790c cells was performed as described above and analyzed by western blotting.

### Statistical analysis

All data are represented as mean ± SD and were analyzed using GraphPad Prism version 5.0. Statistical differences were assessed by two-tailed unpaired *t*-test between groups. *p* < 0.05 was considered to indicate a statistically significant.

## Results

### Rv0790c facilitated mycobacterial survival both *in vitro* and *in vivo*

**Figure 1:**
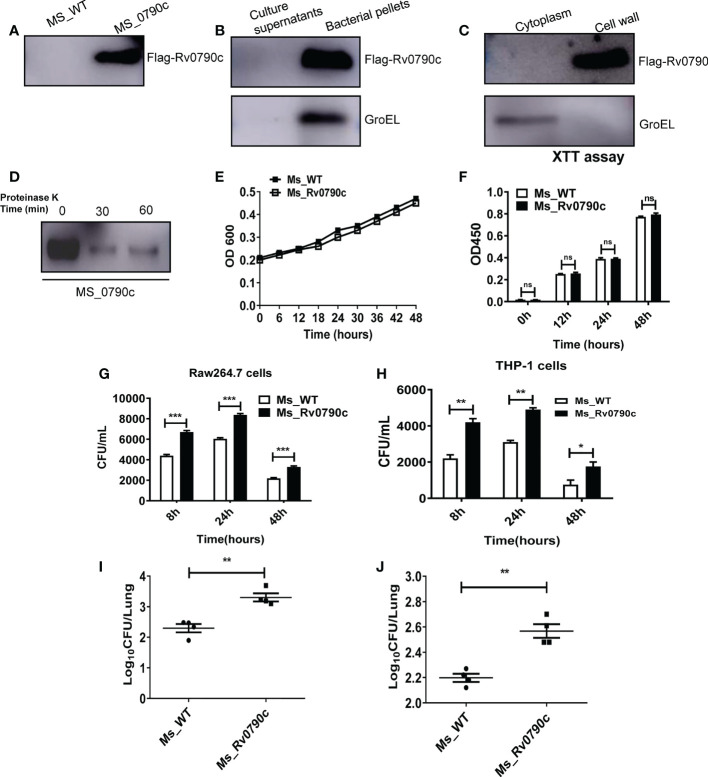
Rv0790c potentiated mycobacterial survival in infected cells and mice. **(A)** The expression of Rv0790c in *MS_Rv0790c*. Equal amounts of *MS_Rv0790c* and *MS_WT* were collected and detected by western blotting using a mouse anti-FLAG antibody. **(B)** Rv0790c was not presented in *MS_Rv0790c* culture medium. The Rv0790c expression was detected in *MS_Rv0790c* culture filtrates and bacteria pellets by western blotting. The GroEL served as a cytosol marker control. **(C)** Rv0790c was associated with cell wall of *MS_Rv0790c*. The cell wall and cytosol-membrane fractions of *MS_Rv0790c* were separated, and western blotting was performed using anti-Flag antibody for the detection of Rv0790c. **(D)** Rv0790c was reduced after proteinase K digestion. Equal amounts *MS_Rv0790c* were incubated with proteinase K (100μg/mL) for the indicated time points and the cell lysates were analyzed for the level of Rv0790c using western blotting. **(E)** The growth curves of *MS_WT* and *MS_Rv0790c*. *MS_WT* and *MS_Rv0790c* strains were grown in Luria-Bertani medium supplemented with 0.05% Tween-80, the OD600 wavelength were determined as the bacterial density at an interval of six hours. **(F)** The proliferation of *MS_WT* and *MS_Rv0790c*. Equal amounts of *MS_Rv0790c* and *MS_WT* was seeded on 96-well plate for XTT bacteria proliferation assay in different times. **(G)** Rv0790c promoted mycobacterial survival in RAW264.7 cells and **(H)** THP-1 cells. RAW264.7 cells or THP1 cells were infected with *MS_Rv0790c* and *MS_WT* (MOI=20). The cells were harvested at indicated time points and the bacillary burden was determined by CFU assay. Rv0790c enhanced mycobacterial survival in mice. Mice (n=4 per group) were **(I)** intranasally or **(J)** intraperitoneally infected with *MS_WT* and *MS_Rv0790c* (2×10^7^ CFU/mice). The bacillary burden in lung tissues were determined by CFU assay. ns, nonsignificant; *p < 0.05; **p < 0.01; ***p < 0.001. Data are shown as mean ± SD and the representative of three independent experiments.

*MS* is an ideal model for studying the functions of *Mtb* genes. According to the Tuberculist Web, we noticed that *Mtb* encoded Rv0790c homologous gene was not present in the genome of *MS*. Thus, to evaluate the role of Rv0790c, we generated *MS_Rv0790c* that could exogenously express *Mtb* Rv0790c in *MS*. The expression of Rv0790c in *MS_Rv0790c* was confirmed *via* western blotting (**Figure 1A**), and we found that Rv0790c was not secreted by *MS_Rv0790c* (**Figure 1B**). The cell wall and membrane-cytosol fractions indicated that the subcellular localization of Rv0790c was in the *MS_Rv0790c* cell wall (**Figure 1C**). Additionally, the protein level of Rv0790c was significantly reduced after proteinase K digestion, suggesting that Rv0790c may be located on the surface of the bacterial cell wall, which is accessible to proteinase K (**Figure 1D**). The growth curve of *MS_Rv0790c* in Luria-Bertani medium indicated no significant difference between the growth of *MS_Rv0790c* and *MS_WT* at different time points (**Figures 1E, F**), suggesting the expression of Rv0790c in recombinant *MS_Rv0790c* did not affect bacillary growth. Furthermore, we examined whether Rv0790c could affect bacillary survival in infected RAW264.7 or THP-1 cells. The cells were infected with *MS_Rv0790c* or *MS_WT* at a MOI of 20 and harvested at different time points for the detection of intracellular bacillary burden, using the CFU assay. We observed that *MS_Rv0790c* had a higher rate of survival in both infected cells than *MS_WT* (**Figures 1G, H**). To confirm whether Rv0790c could enhance mycobacterial survival *in vivo*, C57BL/6 mice were intranasally infected with *MS_WT* and *MS_Rv0790c*. The bacillary burden in the lungs of *MS_Rv0790c* infected mice was significantly higher compared with that of the *MS_WT* infected mice 6 days post-infection (**Figure 1I**). Similar results were obtained when infection was induced by intraperitoneal injection (**Figure 1J**). Taken together, these results demonstrate that Rv0790c facilitates bacillary survival *in vitro* and *in vivo*.

### Knockout of Rv0790c impeded *H37Rv* survival in infected macrophages

**Figure 2:**
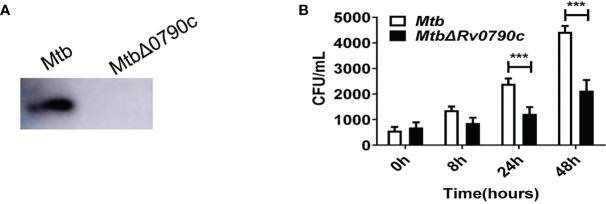
Rv0790c enhanced *H37Rv* survival in infected macrophages. **(A)** The knockout of Rv0790c in *Mtb* strain *H37Rv*. The Rv0790c expression was detected in *H37RvΔRv0790c* by western blotting using anti-Rv0790c homemade mouse antibody. **(B)** The survival of *H37RvΔRv0790c* decreased in infected RAW264.7 cells. The RAW264.7 cells were infected with *H37Rv* or *H37RvΔRv0790c* (MOI=10). The cells were harvested at indicated time points and the bacterial burden was determined by CFU assay. ***p < 0.001. Data are shown as mean ± SD and are representative of three independent experiments.

To further explore the role of Rv0790c during *Mtb* infection, we acquired the Rv0790c knockout *Mtb* strain (*H37Rv△Rv0790c*, Gene optimal Inc, Shanghai). Deletion of Rv0790c in *H37Rv△Rv0790c* was confirmed by western blotting (**Figure 2A**). We compared the intracellular bacterial survival of *H37Rv* and *H37Rv△Rv0790c* in macrophages. RAW264.7 cells were infected with *H37Rv* or *H37Rv△Rv0790c*, and the bacterial load was determined by the CFU assay. The number of CFUs in *H37Rv*-infected macrophages was higher than that in *H37Rv△Rv0790c-*infected RAW264.7 cells, especially 24h and 48h post-infection (**Figure 2B**). Consistent with **Figures 1G, H**, an increased bacterial load was observed when Rv0790c was exogenously expressed in *MS*. Overall, these results further demonstrate that Rv0790c could promote *Mtb* survival in infected macrophages.

### Rv0790c promoted mycobacterial survival were not depend on inhibition of inflammation or induction of apoptosis

**Figure 3:**
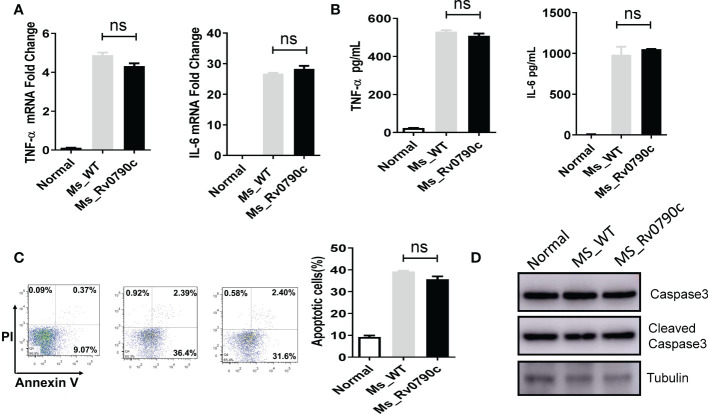
The impact of Rv0790c on inflammatory response and apoptosis in mycobacterial-infected macrophages. RAW264.7 cells were infected with *MS_WT* or *MS_Rv0790c* (MOI=10) for 24hrs. **(A)** The fold change of TNF-α and IL-6 mRNA level. **(B)** The concentration of TNF-α and IL-6 in culture medium analyzed by ELISA. **(C)** The apoptosis was detected in infected cells. RAW264.7 cells were infected with *MS_WT* or *MS_Rv0790c* (MOI=10) for 48hrs. The percentages of total apoptotic cells (Annexin V positive) were determined by flow cytometry. **(D)** RAW264.7 cells were infected with *MS_WT* or *MS_Rv0790c* (MOI=10) for 24 hrs. The cellular apoptosis was detected using cleaved-caspase3 antibody in western blotting. ns, nonsignificant. Data are shown as mean ± SD and the representative of three independent experiments.

Macrophages have been shown to play essential roles in inflammatory responses to neutralize invading pathogens (Lam et al., 2017; Fu et al., 2021). Thus, we investigated whether the promotion of mycobacterial survival mediated by Rv0790c is associated with inflammation in infected macrophages. RAW264.7 cells were infected with *MS_WT* and *MS_Rv0790c*, and the levels of the inflammatory cytokines, TNF-α and IL-6 were detected. Surprisingly, inflammatory cytokine levels in *MS_Rv0790c*- and *MS_WT*-infected cells were not significantly different (**Figures 3A, B**). It has also been reported that intracellular bacteria can modulate the host apoptotic apparatus for optimal replication and dissemination (Hacker, 2018). The effect of Rv0790c on macrophage apoptosis was detected using flow cytometry and western blotting. As shown in **Figure 3C**, Annexin V staining of infected cells indicated similar levels of apoptosis between *MS_Rv0790c*- and *MS_WT*-infected RAW264.7 cells. Further, there was no difference in cleaved-Capase3 levels when examined by western blotting (**Figure 3D**). These results suggest that the promotion of mycobacterial survival mediated by Rv0790c may not depend on the inhibition of inflammation or apoptosis in infected cells.

### Rv0790c inhibited the cellular autophagy at its early stage

**Figure 4:**
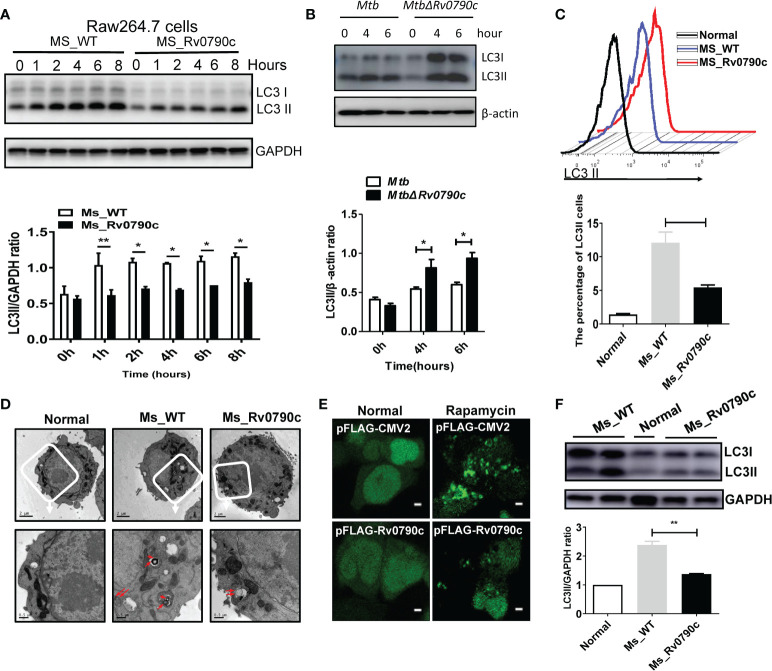
Rv0790c inhibited the autophagy in infected cells and mice. **(A)** *MS_Rv0790c* inhibited the expression of LC3II in infected RAW264.7 cells. RAW264.7 cells were infected with *MS_WT* or *MS_Rv0790c* (MOI=10) and the level of IC3II at indicted times was detected using western blotting. The gray intensity of bands in the SDS-PAGE Gel was measured by ImageJ software. The relative LC3-II expression compared with GAPDH was calculated in the low panel. **(B)** Rv0790c knockout promoted cellular autophagy in *H37Rv△Rv0790c* infected RAW264.7 cells. The RAW264.7 cells were infected with H37Rv or H37RvΔRv0790c (MOI=10) for 4 or 6 hours, the expression level of LC3-II was detected by western blotting. The gray intensity of bands in the SDS-PAGE Gel was measured by ImageJ software. The relative LC3-II expression compared with β-actin was calculated in the low panel. **(C)** FACS analysis of LC3II expression in *MS_Rv0790c* infected RAW264.7 cells. RAW264.7 cells were infected with *MS_WT* or *MS_Rv0790c* (MOI=10) for 4hrs. The cells were then incubated with anti-LC3 antibody and anti-rabbit IgG488 for FACS analysis. **(D)** Rv0790c inhibited the production of autophagic vacuoles in infected RAW264.7 cells. RAW264.7 cells were infected with *MS_WT* or *MS_Rv0790c* (MOI=10) for 4hrs and then assay for TEM. The autophagic vacuoles in up panel (white box) were indicated in red arrows (down panel). Scale bar: 2µM, upper panel; 0.5µM, lower panel. **(E)** HEK293T cells were co-transfected with mCherry-GFP-LC3 and Flag-Rv0790c for 36hrs and then treated with rapamycin (100nM) for 2hrs. The autolysosome (red) was detected by confocal laser scanning microscopy. Scale bar: 2.5 µM **(F)** Rv0790c inhibited autophagy in lungs of infected mice. C57BL/6 mice (n=4) were intranasally infected with *MS_WT* or *MS_Rv0790c* (2×10^7^) for 6 days. The LC3II expression in lungs of infected mice was detected by western blotting. *p < 0.05; **p < 0.01. Data are shown as mean ± SD and the representative of three independent experiments.

Autophagy is important for macrophages to clear *Mtb* out of the cellular systems (Liu et al., 2017; Paik et al., 2019). Hence, we assessed the role of Rv0790c in autophagy modulation. RAW264.7 cells were infected with *MS_Rv0790c* and *MS_WT*, and the expression level of LC3-II was examined by western blotting. Interestingly, *MS_Rv0790c*-infected RAW264.7 cells exhibited less autophagy, as indicated by the lower expression of LC3-II protein compared to that in *MS_WT*-infected cells (**Figure 4A**). A similar phenomenon was also observed in Rv0790c deficient *Mtb H37Rv△Rv0790c* compared with parental strain *H37Rv*, confirming the inhibitory effects of Rv0790c on autophagy (**Figure 4B**). Furthermore, exogenously stably expressing Rv0790c was sufficient to inhibit autophagy in rapamycin-treated RAW-Rv0790c cells, suggesting that Rv0790c was a potent autophagy inhibitor even without the presence of mycobacterial infection (**Supplementary data 1**. 4B). Flow cytometry assay also indicated that the percentage of LC3II^+^ cells decreased in *MS_Rv0790c*-infected RAW264.7 cells (**Figure 4C**). The number of autophagic vacuoles was reduced in *MS_Rv0790c*-infected cells, as measured by transmission electron microscopy (TEM) (**Figure 4D**). In addition, HEK293T cells co-transfected with mCherry-GFP-LC3 and Flag-Rv0790c showed fewer red spots compared with that of the vector control, with or without rapamycin (**Figure 4E**). To examine whether Rv0790c inhibits autophagy *in vivo*, C57BL/6 mice were intranasally infected with *MS_Rv0790c* or *MS_WT*. As shown in **Figure 4F**, LC3-II expression was significantly decreased in the lungs of *MS_Rv0790c*-infected mice, compared to that in the lungs of *MS_WT*-infected mice. Together, these results demonstrate that Rv0790c inhibited autophagy during mycobacterial infection both *in vitro* and *in vivo*.

**Figure 5:**
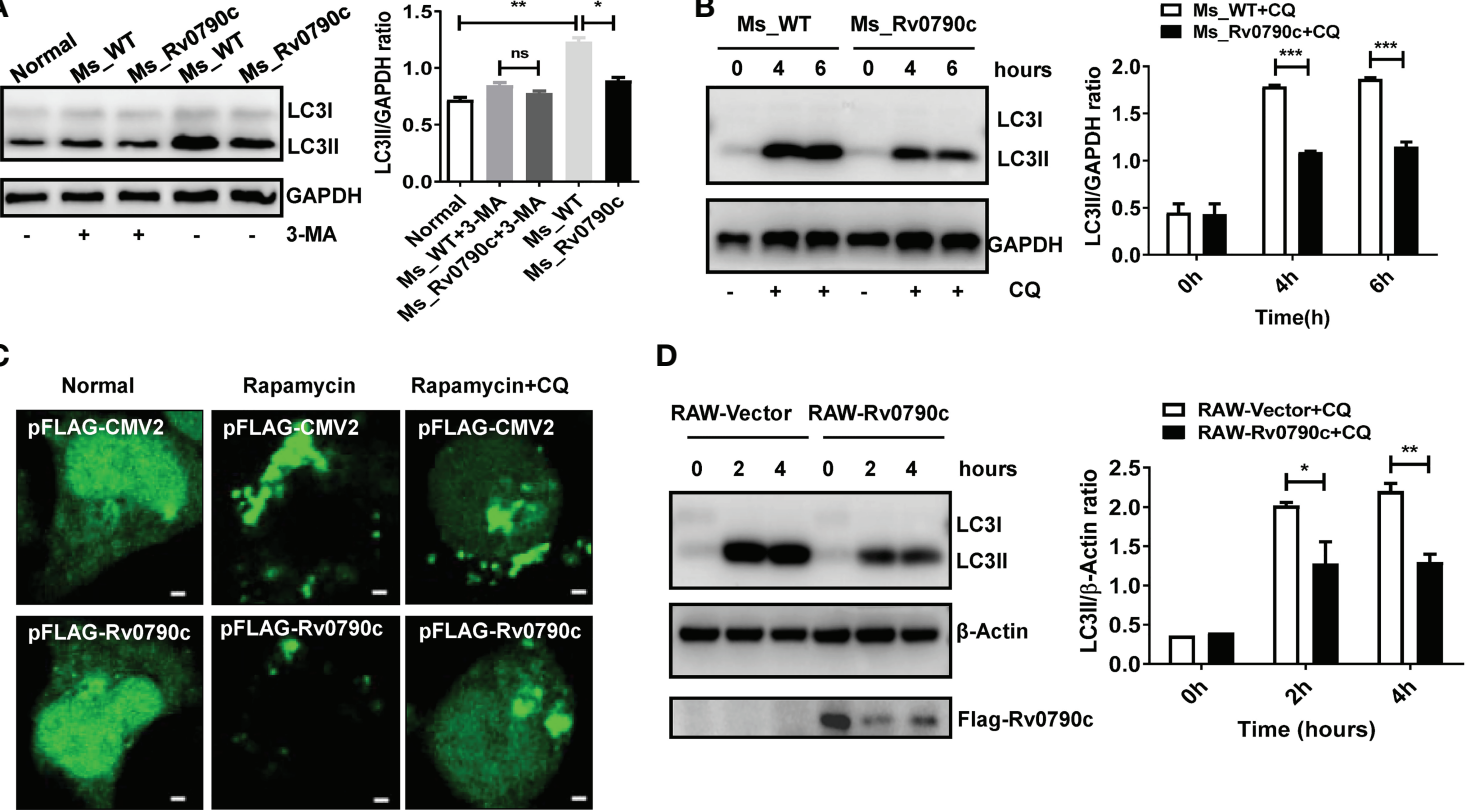
Rv0790c suppressed autophagy at its early stage. **(A)** The RAW264.7 cells were infected with *MS_WT* or *MS_Rv0790c* (MOI=10) and treated with 3-MA (10mM) for 4h. The expression level of LC3II was detected by western blotting. **(B)** The RAW264.7 cells were infected with *MS_WT* or *MS_Rv0790c* (MOI=10) and treated with CQ (100nM) as indicated times. The expression level of LC3II was detected by western blotting. **(C)** Rv0790c inhibited the production of LC3 puncta even in the presence of CQ. The HEK293T cells were co-transfected with GFP-LC3 and Flag-Rv0790c, and treated with rapamycin (100nM) and CQ (100nM). The LC3 puncta were detected by confocal laser scanning microscopy scale bars:0.5μm. **(D)** Rv0790c inhibited autophagy in RAW-Rv0790c cells. The RAW-Vector and RAW-Rv0790c cells were treated with rapamycin (100nM) plus CQ (100nM) as indicated times. The cells were harvested and the expression level of LC3II was detected by western blotting. ns, nonsignificant; *p < 0.05; **p < 0.01; ***p < 0.001. Data are shown as mean ± SD and the representative of three independent experiments.

Autophagy is a conserved biological process, including initiation, elongation, maturation of autophagosomes, and their eventual fusion with lysosomes (Singh and Subbian, 2018; Wang et al., 2019). To further determine which stage of Rv0790c affects autophagy, 3-Methyladenine (3-MA) (inhibiting the initiation of autophagy) and CQ (inhibiting autophagosome-lysosome fusion) were applied. Interestingly, the expression level of LC3-II did not decrease in *MS_Rv0790c*-infected macrophages treated with 3-MA (**Figure 5A**). However, unlike 3-MA treatment, the expression level of LC3-II protein was still decreased in *MS_Rv0790c*-infected macrophages, compared to that in the control group after CQ treatment (**Figure 5B**). Immunofluorescence also confirmed that CQ treatment did not affect LC3 puncta formation in Flag-Rv0790c transfected HEK293T cells (**Figure 5C**). Consistently, CQ treatment did not relieve the Rv0790c-induced autophagy inhibition in RAW-Rv0790c cells (**Figure 5D**). These data suggest that Rv0790c suppresses autophagy at its early stage.

### Rv0790c interaction with mTOR

**Figure 6:**
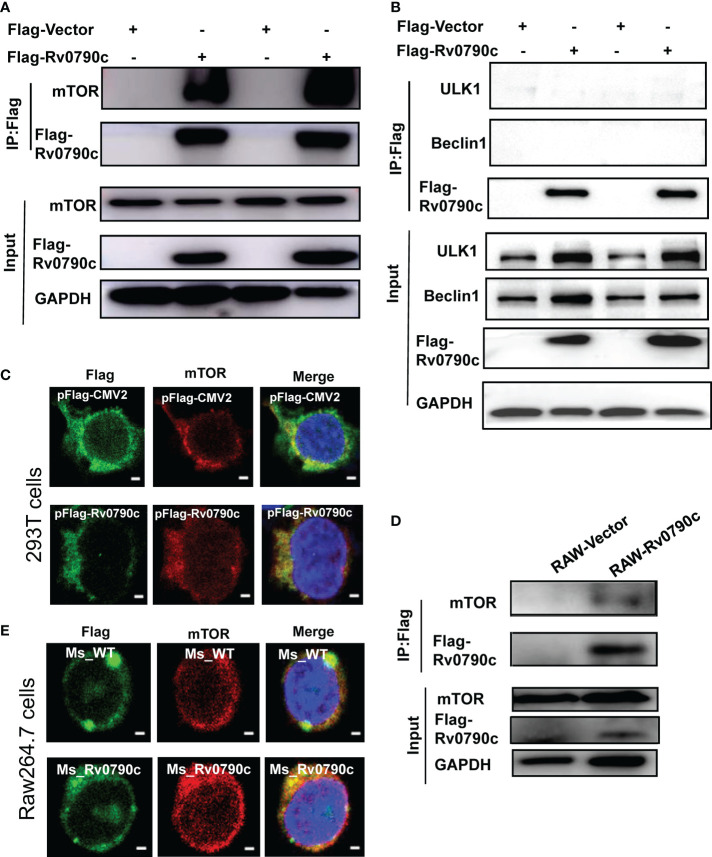
Rv0790c interaction with mTOR. HEK293T cells were transfected with Flag-Rv0790c or vector control. The cell extracts were immunoprecipitated with anti-Flag-beads and analyzed by the indicated antibodies **(A)** mTOR; **(B)** ULK1 and Beclin1 using western blotting. **(C)** RAW264.7 cells stably expressing Flag-Rv0790c were harvested and the cell extracts were immunoprecipitated with anti-Flag-beads and analyzed by the indicated antibodies using western blotting. **(D)** The co-localization of mTOR and Flag-Rv0790c in HEK293T cells. HEK293T cells were transfected with Flag-Rv0790c or control vector for 36hrs and then stained with anti-Flag (Alexa Fluor 488; green) and anti-mTOR (Alexa Fluor 633; red) antibodies for IF assay. Scale bars:0.5μm; **(E)** The co-localization of mTOR and Flag-Rv0790c in *MS_Rv0790c* infected Raw264.7cells. Raw264.7 cells were infected with *MS_WT* or *MS_Rv0790c* (MOI=10) for 4h. The cells were then stained using anti-Flag (Alexa Fluor 488; green) and anti-mTOR (Alexa Fluor 633; red) antibodies for IF assay. Scale bars:0.5μm. Data are shown as mean ± SD and are representative of three independent experiments.

Autophagy is initiated by mTORC1 complex inhibition, following ULK complex and PI3K complex assembly (Galluzzi and Green, 2019). To investigate how Rv0790c modulates autophagy initiation, we hypothesized that Rv0790c inhibits autophagy initiation by interacting with autophagy-related proteins. To this end, we screened several proteins, including mTOR, ULK-1, and Beclin1, which are related to autophagy initiation, by immunoprecipitation assay. As shown in **Figure 6A**, Rv0790c co-immunoprecipitated with mTOR, but not with the other proteins (ULK-1 and Beclin1) in Flag-Rv0790c- transfected HEK293T cells (**Figure 6B**). Immunofluorescence assay showed that Rv0790c and mTOR co-localized in Flag-Rv0790c transfected HEK293T cells (**Figure 6C**). Co-IP also showed that Rv0790c interacted with mTOR in Rv0790c stable expressing RAW-Rv0790c cells (**Figure 6D**). In addition, Rv0790c expressed by *MS_Rv0790c* also co-localized with mTOR when Raw264.7 cells were infected with *MS_Rv0790c* (**Figure 6E**). These results suggest that Rv0790c does interact with mTOR.

### mTOR activity enhanced by Rv0790c interaction

**Figure 7:**
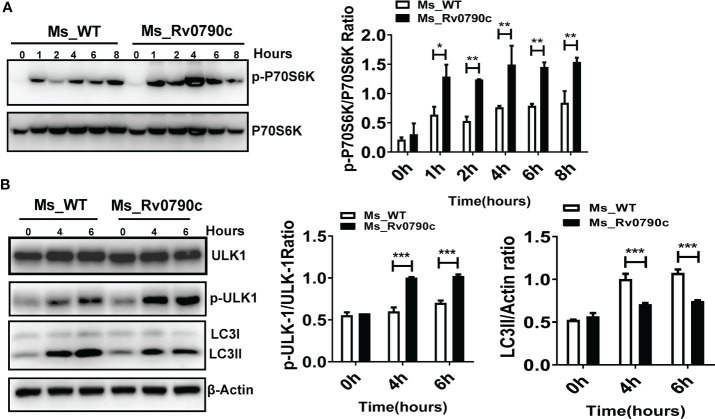
mTOR activity enhanced by Rv0790c. **(A)** Rv0790c enhanced mTOR activity in infected macrophages. RAW264.7 cells were infected with *MS_WT* or *MS_Rv0790c* (MOI=10) at the indicated time points. The expression level of phospho-P70S6K and total-P70S6K were analyzed by western blotting. **(B)** The activity of ULK, a downstream target molecule of mTOR, was inhibited in *MS_Rv0790c* infected macrophages. RAW264.7 cells were infected with *MS_WT* or *MS_Rv0790c* (MOI=10) at the indicated time points. The expression level of phospho-ULK-1^ser757^, total-ULK-1 and LC3II were analyzed by western blotting. *p < 0.05; **p < 0.01; ***p < 0.001. Data are shown as mean ± SD and are representative of three independent experiments.

Since mTOR is a master regulator of cellular autophagy (Deleyto-Seldas and Efeyan, 2021), we detected the phosphorylation level of P70S6 kinase, an mTOR downstream effector, as an indicator of mTOR activity, in the presence of Rv0790c. The level of phosphorylated P70S6 kinase at Thr^389^ in *MS_Rv0790c*-infected macrophages was higher than that in *MS_WT* infected-cells, indicating that Rv0790c potentially enhanced mTOR activation (**Figure 7A**). High level of mTOR activity also disrupts autophagy initiation by phosphorylating ULK-1 at ser^757^ (Kim et al., 2011; Ravenhill et al., 2019). We further measured the phosphorylation level of ULK-1^ser757^. Indeed, the phosphorylation level of ULK-1^ser757^ in *MS_Rv0790c*-infected RAW264.7 cells was higher than that in *MS_WT*-infected cells (**Figure 7B**). Consistently, we observed that the expression of LC3-II was reduced in *MS_Rv0790c*-infected cells (**Figure 7B**). Together, these results imply that Rv0790c impaired autophagy initiation by enhancing mTOR activity.

## Discussion

Resident alveolar macrophages act as first responders in the early stages of *Mtb* infection (Cohen et al., 2018). Multiple cellular processes, including phagocytosis, autophagy, apoptosis, and inflammasome formation, have been adopted by alveolar macrophages to resist the invading *Mtb* (Shim et al., 2020). *Mtb* also displays an extraordinary ability to evade components of the innate immunity and successfully inhabit the host through long-term evolution (Liu et al., 2017), which could be partially attributed to the abundance of virulence factors released by *Mtb* (Zhang et al., 2018; Qiang et al., 2019; Ali et al., 2020). Rv0790c is predicted to be a 27 kDa conserved hypothetical protein of *Mtb*, with studies indicating that Rv0790c expression is elevated in *Mtb*-infected macrophages, and that it correlates with moyR, a potential monooxygenase regulator (Parvati Sai Arun et al., 2018; Abeywickrama and Perera, 2021). Genome-wide mutant screening implied that Rv0790c might be one of the candidates affecting *Mtb* growth (Rengarajan et al., 2005). However, little is known about Rv0790c. In an attempt to understand Rv0790c, we found that it inhibits autophagy initiation but not inflammation and apoptosis, and further facilitates bacterial survival in *Mtb* infected-macrophages, which has not been previously characterized.

Autophagy is a highly conserved degradation system that participates in the elimination of intracellular pathogens as well as maintaining cellular homeostasis (Pellegrini et al., 2021). Compared to *Mtb* and *BCG*, *MS* induces a stronger autophagy response (Feng et al., 2020) and *Mtb* strains isolated from different clinical patients have different autophagy-inducing abilities, leading to different bacterial loads and disease severities (Li et al., 2016). Disruption of the autophagy pathway by deleting or downregulating autophagy core proteins, such as LC3B, Atg5, and Atg7, can promote mycobacterial survival in macrophages and mice (Bonilla et al., 2013; Sivangala Thandi et al., 2020), indicating that macrophage autophagy is a crucial cell process that restricts *M.tb* survival. Here, overexpression of Rv0790c in *MS* clearly showed that Rv0790c inhibited autophagy, but not inflammation or apoptosis, in upregulated macrophages. Furthermore, compared to that of the control group of CQ treatment, the level of LC3-II was no longer decreased in *MS_Rv0790c*-infected macrophages treated with 3-MA, suggesting that Rv0790c inhibits the early stage of autophagy. This is different from previously reported *Mtb* encoding proteins that function in autophagy that most likely inhibit the fusion of autophagosomes and lysosomes, which is the late stage of autophagy. For instance, *Mtb* PhoP and ESAT-6 block autolysosome formation by preventing Rab7 recruitment (Chandra et al., 2015). Further, *Mtb* PknG inhibits phagosome-lysosome fusion by targeting the Rab7l1 signalling pathway (Pradhan et al., 2018). Although there is no evidence, inhibition at the initiation of autophagy seems more vigorous than that at the late stage. We believe that Rv0790c may play a role in the early stages of mycobacterial infection. According to our results, Rv0790c is deposited on the bacterial cell wall. Therefore, it is reasonable to conclude that Rv0790c executes its function of autophagy inhibition for bacterial maintenance in infected macrophages immediately after entry. While infection continues, it might become more efficient to inhibit autophagy at the late stage by other *Mtb* proteins for the replication of progeny mycobacteria because studies have shown that autophagosomes, not autolysosomes, provide a sheltered niche for bacterial survival and replication (Ge et al., 2021). Hence, we believe that although *Mtb* has different effector proteins that resist host autophagy degradation, these effectors may work at different stages for the most efficient bacterial survival during infection.

mTOR is a highly conserved serine/threonine protein kinase, with a molecular mass of 289 kDa. Activation of mTOR signaling promotes gene expression and protein synthesis (Liu and Sabatini, 2020). The role of mTOR in autophagy regulation has been well-documented in many studies (Kim et al., 2011; Shang and Wang, 2011; Munson and Ganley, 2015). Once mTOR activity is suppressed, downstream autophagy-related genes are potently activated for the induction of autophagy, which balances cell survival under stress conditions (Nowosad et al., 2020; Fong et al., 2021; Shao et al., 2021). We found that Rv0790c strongly interacted with mTOR and interestingly, this interaction increased mTOR activity as observed by the increased phosphorylation of mTOR downstream molecules p70S6K^Thr389^ and ULK-1^ser757^, leading to the suppression of autophagy initiation in mycobacteria-infected macrophages. It has been reported that binding of mTOR to RAG GTPases and Rheb results in enhanced mTOR activity (Sancak et al., 2008). Additionally, mTOR activity can be enhanced by TRAF6-mediated K63 ubiquitination (Linares et al., 2013). It is possible that Rv0790c acts as a bridge that enhances the binding capacity of mTOR to these components in the complex. However, the exact mechanism by which Rv0790c enhances mTOR activity requires further elucidation as well as the Th1 cytokine IFN-γ and Th2 cytokines IL-4, IL-13, because these cytokines were also shown the ability to modulate autophagy in *Mtb* infection (Ghadimi et al., 2010; Yang et al., 2021). Our results are in line with other studies that have also found that *Mtb* escapes autophagic degradation by regulating mTOR signaling. For example, a study showed how *Mtb* calcium pump CtpF mediates Ca^2+^ efflux and inhibits mTOR-dependent autophagy in macrophages (Garg et al., 2020). Further, *Mtb* Eis potentially inhibits macrophage autophagy by activating the Akt/mTOR/P70S6K pathway (Duan et al., 2016).

*Ms* is a good model for the study of *Mtb* protein functions (Bashiri and Baker, 2015) and we found that the bacillary burden in the lungs of MS_Rv0790c infected mice was significantly higher compared with that of the MS_WT infected mice 6 days post-infection. To further prove the role of Rv0790c in the outcome of *Mtb* infection, we deleted the Rv0790c in *Mtb*. Deletion of Rv0790c significantly enhances autophagy and reduces the survival of *Mtb* in macrophages. However, we haven’t carefully examined the Rv0790c functions within H37Rv_Δ0790c infected mice due to the safety guideline, which might be a flaw of this study. Nevertheless, we also detected the transcriptional level of Rv0790c in clinical isolates and found that they showed differential expression compared with the standard virulent strain of *H37Rv*, suggesting that Rv0790c is preferentially expressed in virulent mycobacteria and may be used as an indicator of clinical disease severity.

In summary, we identified a novel *Mtb* factor, Rv0790c, which inhibits cellular autophagy to evade host clearance against mycobacterial infection. Rv0790c inhibits autophagy by directly interacting with mTOR and promoting its activity, thereby facilitating *Mtb* survival in infected macrophages. These findings highlight the potential of mTOR-mediated autophagy as a host-directed therapeutic drug.

## Data availability statement

The original contributions presented in the study are included in the article/**Supplementary Material**. Further inquiries can be directed to the corresponding author.

## Ethics statement

The animal study was reviewed and approved by the ethics committee of Soochow University.

## Author contributions

JF performed all experiments and analyzed the data. SX and CD designed the experiments and wrote the manuscript. All authors have given final approval of the version to be published and agree to be accountable for all aspects of the work. All authors contributed to the article and approved the submitted version.

## Funding

This work was supported by grants from the National Natural Science Foundation of China (31970844, 32170927,31870867, 32170148), The National Science and Technology Key Project (2018ZX10731301-004-003). Jiangsu Provincial Innovative Research Team and the Priority Academic Program Development of Jiangsu Higher Education Institutions (PAPD).

## Acknowledgments

We thank Dr. Wei Zhang for the critical technical support and discussion in the study.

## Conflict of interest

The authors declare that the research was conducted in the absence of any commercial or financial relationships that could be construed as a potential conflict of interest.

## Publisher’s note

All claims expressed in this article are solely those of the authors and do not necessarily represent those of their affiliated organizations, or those of the publisher, the editors and the reviewers. Any product that may be evaluated in this article, or claim that may be made by its manufacturer, is not guaranteed or endorsed by the publisher.
