# Supplementary figures and images for: Mycobacterium tuberculosis Rv0790c inhibits the cellular autophagy at its early stage and facilitates mycobacterial survival
Source: Front Cell Infect Microbiol. 2022 Nov 9;12:1014897. doi: 10.3389/fcimb.2022.1014897 (PMC9683476; doi:10.3389/fcimb.2022.1014897)

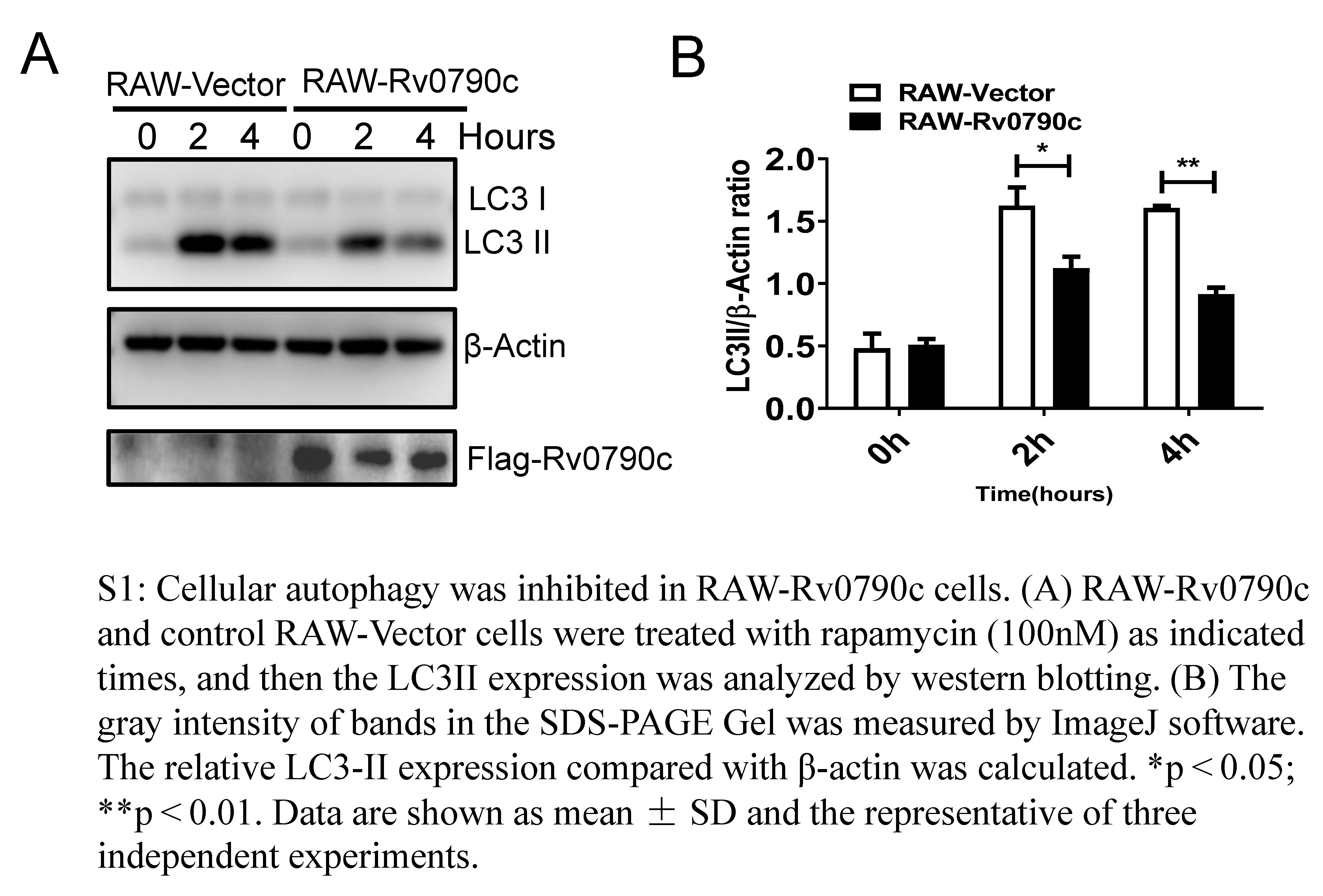

Supplement: Supplementary file 1 [file Image_1.jpeg]
